# Exogenous Ketone Supplements Improved Motor Performance in Preclinical Rodent Models

**DOI:** 10.3390/nu12082459

**Published:** 2020-08-15

**Authors:** Csilla Ari, Cem Murdun, Craig Goldhagen, Andrew P. Koutnik, Sahil R. Bharwani, David M. Diamond, Mark Kindy, Dominic P. D’Agostino, Zsolt Kovacs

**Affiliations:** 1 Department of Psychology, Behavioral Neuroscience Research Laboratory, University of South Florida, Tampa, FL 33620, USA; sahilbharwani2692@gmail.com (S.R.B.); ddiamond@usf.edu (D.M.D.); 2 Ketone Technologies, Tampa, FL 33612, USA; ddagosti@usf.edu; 3 Department of Molecular Pharmacology and Physiology, Laboratory of Metabolic Medicine, Morsani College of Medicine, University of South Florida, Tampa, FL 33612, USA; biocem@gmail.com (C.M.); cgoldhagen@usf.edu (C.G.); akoutnik@usf.edu (A.P.K.); 4 Institute for Human and Machine Cognition, Ocala, FL 34471, USA; 5 Department of Pharmaceutical Sciences, College of Pharmacy, University of South Florida, Tampa, FL 33612, USA; kindym@health.usf.edu; 6 James A. Haley VA Medical Center, Tampa, FL 33612, USA; 7 Shriners Hospital for Children, Tampa, FL 33612, USA; 8 Savaria Department of Biology, ELTE Eötvös Loránd University, Savaria University Centre, Károlyi Gáspár tér 4., 9700 Szombathely, Hungary; zskovacsneuro@gmail.com

**Keywords:** ketone ester, ketogenic diet, ketone salt, MCT, rotarod, R-βHB

## Abstract

Nutritional ketosis has been proven effective for neurometabolic conditions and disorders linked to metabolic dysregulation. While inducing nutritional ketosis, ketogenic diet (KD) can improve motor performance in the context of certain disease states, but it is unknown whether exogenous ketone supplements—alternatives to KDs—may have similar effects. Therefore, we investigated the effect of ketone supplements on motor performance, using accelerating rotarod test and on postexercise blood glucose and *R*-beta-hydroxybutyrate (*R*-βHB) levels in rodent models with and without pathology. The effect of KD, butanediol (BD), ketone-ester (KE), ketone-salt (KS), and their combination (KE + KS: KEKS) or mixtures with medium chain triglyceride (MCT) (KE + MCT: KEMCT; KS + MCT: KSMCT) was tested in Sprague-Dawley (SPD) and WAG/Rij (WR) rats and in GLUT-1 Deficiency Syndrome (G1D) mice. Motor performance was enhanced by KEMCT acutely, KE and KS subchronically in SPD rats, by KEKS and KEMCT groups in WR rats, and by KE chronically in G1D mice. We demonstrated that exogenous ketone supplementation improved motor performance to various degrees in rodent models, while effectively elevated *R*-βHB and in some cases offsets postexercise blood glucose elevations. Our results suggest that improvement of motor performance varies depending on the strain of rodents, specific ketone formulation, age, and exposure frequency.

## 1. Introduction

Motor impairment can be caused by injury or degeneration to the motor cortex, premotor cortex, motor tracts, or associated pathways in the cerebrum, cerebellum, or neuromuscular junction. These pathological changes have been observed in a variety of neurological conditions, such as Alzheimer’s Disease (AD), Huntington’s Disease (HD), Parkinson’s Disease (PD), Amyotrophic Lateral Sclerosis (ALS), Glucose 1 Deficiency Syndrome (G1D), or those who have suffered a cerebral vascular accident (CVA) stroke, and in patients with traumatic brain injuries (TBI) [1,2,3,4,5,6]. These neurological conditions present with metabolic impairment, neuroinflammation, and other related molecular characteristics. Nutritional ketosis significantly improved outcomes and motor function in HD, PD, ALS, G1D, and AD through various mechanisms, such as reduced proinflammatory cytokines, decreased mitochondrial damage, and preservation of cellular bioenergetics. These results suggest that the mechanism of action for ketone bodies to enhance/preserve motor performance is multifactorial, but has significant overlap between biological pathways [1,2,3,4,5,6,7,8,9]. However, the predominant mechanism of action of nutritional ketosis on motor performance is largely unknown, as is the optimal method of administration.

The ketogenic diet (KD) was designed to induce nutritional ketosis, initially employed for its metabolic benefits and ability to treat epilepsy [10]. Physiologically, the metabolic response to a KD closely resembles the insulin suppression associated with fasting if total calories and ketogenic ratios (3:1 to 4:1 ratio, by weight, of fat to a combination of protein and carbohydrates) are maintained [5]. Despite the efficacy of the KD clinically, patient compliance can be low due to the strict nutritional requirements, fat intolerance, or potential complications and side effects [11,12]. Subjects report difficulty maintaining ketosis as excess consumption of carbohydrates or protein can rapidly shift the body back towards glycolysis and inhibit ketogenesis [13]. Alternative solutions to achieving nutritional ketosis are needed, and exogenous ketone supplementation has been reported to be an effective alternative to KD in the context of various disease states [11,14,15,16,17,18,19].

Under normal physiological conditions and when adhering to a “traditional” western diet typically rich in carbohydrate, glucose is the primary metabolic fuel in the CNS and skeletal muscle [20]. However, during low glucose bioavailability and hepatic glycogen depletion, free fatty acids are mobilized from adipose tissue and released into the bloodstream to be used directly as fuel or converted to ketone bodies in the liver through the process of ketogenesis to sustain metabolic demands [21]. The primary ketone bodies produced, acetoacetate (AcAc) and R-β-hydroxybutyrate (R-βHB), are subsequently released into the bloodstream and serve as an alternative fuel for most tissues, but especially the central nervous tissue, as long-chain fatty acids cannot readily be used by the brain [22].

Hyperketonemia is the metabolic state characterized by an elevation of blood ketone bodies and is observed in individuals who adhere to a KD or are fasted for extended periods of time [22]. In this state, a large portion of tissue energetic requirements are met via fatty acid oxidation and ketolysis [23]. Under normal physiological conditions, blood ketone levels rarely exceed 0.01 mmol/L and account for less than 3% of total brain metabolism [24]. However, in periods of nutritional ketosis, characterized by blood ketone levels of 0.5–6 mmol/L, ketone bodies cross the blood–brain barrier (BBB) through monocarboxylate class transporters [25] and serve as supplemental fuel accounting for up to 60% of brain energy metabolism [21,26]. Once inside of the cell’s mitochondria, the ketone bodies are converted into Acetyl CoA, enter the TCA cycle, and generate the reduced intermediates (NADH and FADH_2_) needed to sustain ATP production [22].

Supplementation with ketone (ketogenic) supplements, such as ketone ester (KE), has proven effective in achieving a state of nutritional ketosis independent of carbohydrate restriction [14,17,18,27,28]. Ketone supplementation that contains either KE, ketone salt (KS), medium chain triglycerides (MCTs), or their combinations (e.g., KEKS, KEMCT, and KSMCT) has been studied in both animal models and humans. It has been demonstrated that exogenous ketone supplementation is a safe and effective option to induce nutritional ketosis [16,29,30,31,32], and, consequently, evoke alleviating effects of ketosis on motor performance similar to KD [2,4,6,33]. Indeed, it was suggested that administration of KE and MCTs may evoke beneficial effects on motor dysfunction [34,35]. However, it is unknown whether different exogenous ketone supplements and their combinations would increase motor performance in animal models under different conditions associated with motor function impairment. Our previous study also demonstrated that exogenous ketones have a blood glucose lowering effect [27]; however, the hormonal response to exercise can cause stored glucose to be released from the liver and skeletal muscle while temporarily raising blood glucose levels. Thus, the main goal of the present study was to determine if specific ketogenic supplements and/or combinations would improve motor performance in rodents with pathology (Wistar Albino Glaxo/Rijswijk, WAG/Rij/WR rats and Glut1 Deficiency Syndrome/G1D mice) and without pathology (Sprague-Dawley/SPD rats) [36,37] and to test whether they are able to offset the postexercise induced blood glucose elevation. WR rat strain is an accepted model of absence epilepsy with comorbidity of low-grade depression, but without pathological changes in locomotor activity/motor performance [38,39,40], while G1D mice are models of Glut1 Deficiency Syndrome and show motor dysfunction [37,41]. We hypothesized that nutritional ketosis induced by various ketone therapeutics (KD and exogenous ketone supplements) can improve motor performance in rodent models, as defined by latency to fall from the accelerated rotarod and can offset blood glucose elevation in postexercised states.

## 2. Materials and Methods

### 2.1. Animals

Three rodent models were used for the experiments: SPD rats (male, 4 months old and 1 year old, 320–360 g and 540–660 g, respectively, Harlan Laboratories), WR rats (male, 6 months old, 320–360 g, breeding colony, Eötvös Loránd University, Savaria University Centre, Szombathely, Hungary), and G1D mice (male, 3–5 months old, 17–27 g, breeding colony, University of South Florida, Morsani College of Medicine, Tampa, FL, USA) that were housed at either the College of Medicine Animal Facility (Morsani College of Medicine, University of South Florida, Tampa, FL, USA) or at the Savaria Department of Zoology (Eötvös Loránd University, Savaria University Centre, Szombathely, Hungary). Standard laboratory conditions (12:12 h light-dark cycle) were maintained for the animals that were housed in air-conditioned rooms at 22 ± 2 °C in groups of 2–4.

Institutional Animal Care and Use Committee (IACUC; Protocol #0006R) of the University of South Florida (University of South Florida, Tampa, FL, USA) and Hungarian Act of Animal Care and Experimentation (1998. XXVIII. Section 243/1998) and the regulations for animal experimentation in the European Communities Council Directive of 24 November 1986 (86/609/EEC) guidelines were followed during the experimental procedures. Experiments were approved by the Animal Care and Experimentation Committee of the Eötvös Loránd University (Savaria University Centre) and by the National Scientific Ethical Committee on Animal Experimentation (Hungary) under license number VA/ÉBNTF02/85–8/2016. We made all efforts to reduce the number of animals used.

### 2.2. Ketogenic Compounds

Ad libitum access to water and standard rodent chow (standard diet: SD, NIH-31 Rodent Chow; Envigo), or ketogenic rodent food (KD; TD. 10911; Envigo), or SD with ketone supplements (Table 1, and Supplementary Tables S1 and S2) were provided for the animals. The KD used in this study was modified from TD.10787 (Teklad) to remove maltodextrin to make the diet essentially free of carbohydrate. The KD had a 2:1 ratio of n-3 to n-6 fatty acids and a 1.5:1 ratio of fat to protein + carbohydrate. Compared to TD.10787 (common KD formula), TD.10911 had the same % fat (*wt*/*wt*), but it had a higher % kcal from fat, because maltodextrin was removed and replaced with cellulose. KE (R,S 1,3-butanediol-acetoacetate diester) was synthesized by D’Agostino, 2013, as previously described [17]. The KS (Na^+^/K^+^– R,S βHB mineral salt) was mixed into a 50% solution supplying approximately 375 mg/g of pure βHB and 125 mg/g of Na^+^/K^+^ in a 1:1 ratio. KE and KS development and synthesis were performed in collaboration with Savind Inc. Human food-grade MCT oil (~60% caprylic triglyceride/40% capric triglyceride, Now Foods, Bloomingdale, IL, USA) was used for the experiments. KS and KE were mixed with MCT in a 1:1 ratio, resulting the KSMCT and KEMCT combinations, respectively. KE was mixed with KS in a 1:1 ratio to make KEKS. R,S-1,3-butanediol (BD) was purchased from Sigma (Milwaukee, WI, USA).

### 2.3. Exposure Schedule

**Figure 1:**
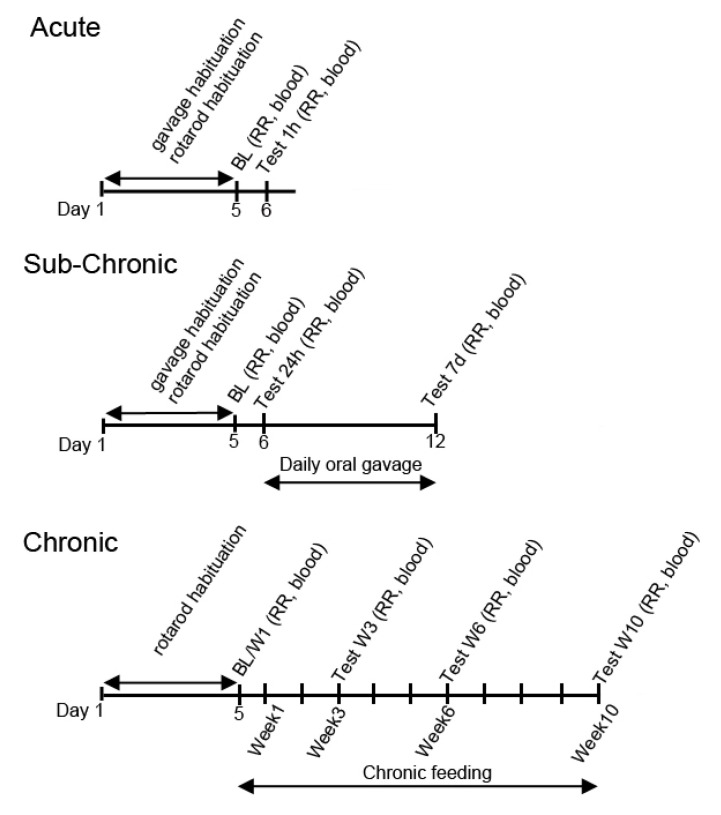
Experimental design for the different exposure schedules (acute, subchronic, and chronic). BL: Baseline measurement; blood: blood draw with glucose and R-βHB measurements; RR: Rotarod test; blood: blood draw with glucose and R-βHB measurements.

For the trials involving SPD and WR rats, intragastric delivery by oral gavage was used. To acclimatize the rodents, each animal was orally gavaged with water for five days prior to treatment (Figure 1). After acclimatization and baseline measurements (5th days), the rats were orally gavaged either once with different exogenous ketone supplements (acute treatment on the 6th day; 5 g/kg for SPD rats) and the effects (motor function and blood measurements) were measured after 30 min^−1^ h, or they were gavaged once daily for 7 days (subchronic treatment between 6th and 12th day; 5 g/kg/day for SPD rats and 2.5 g/kg/day for WR rats) and the effects were recorded after 24 h and after 7 days (Figure 1). In relation to G1D mice with chronic exposure, ketone supplements were administered daily for 10 weeks and the effects were measured after 3, 6, and 10 weeks treatment (Figure 1).

### 2.4. Treatment Groups

One-year-old SPD rats were divided into 6 groups for the acute treatment and were fed with SD and they were administered a single oral gavage. The treatment groups included water (control, *n* = 10), BD (*n* = 8), KE (*n* = 12), KSMCT (*n* = 8), KEKS (*n* = 12), and KEMCT (*n* = 8). Motor performance was evaluated prior to the beginning of dietary treatment (baseline; 5th day of habituation) and 30 min after gavage.

Subchronic experiments used 4-month old SPD rats that were divided into 5 groups and fed with either SD, KD and supplemented with a once-daily oral gavage for 7 days. Treatment groups included control (SD, water gavage, *n* = 11), KD (*n* = 10), KE (*n* = 9), KS (*n* = 9), and KSMCT (*n* = 10). The animals were evaluated for motor performance prior to the beginning of dietary treatment (baseline), 24 h after 1st treatment (KD or gavage), and 1 h after the 7th treatment.

The WR rats with subchronic exposure were divided into 6 groups. They were fed with SD and orally gavaged daily with either water (SD, control; *n* = 9) or KE (*n* = 9), KS (*n* = 9), KSMCT (*n* = 9), KEKS (*n* = 9), KEMCT (*n* = 9) for 7 days. Rotarod test was carried out similarly to subchronically treated SPD rats.

The G1D mice exposed to chronic treatment were split into four groups, SD (control; *n* = 12), KD (*n* = 12), or the SD supplemented with KS (*n* = 12) or KE (*n* = 12) and were treated for 10 weeks. Rotarod test was performed before the beginning of dietary treatment (baseline) and after 3, 6, and 10 weeks of treatment.

### 2.5. Motor Performance Testing

Motor performance was evaluated using the accelerating rotarod test on a RotaRod Rotamex 5 (rotarod; Columbus Inst., Columbus, OH, USA). The rats were placed on the rods of the accelerating rotarod, and the time the animals remained on the rod was measured. The rotarod was set to accelerate from zero to 40 rpm in 180 s. To acclimate the animals to both the equipment and the task, prior to the administration of a test, animals were trained on the rotarod for 5 consecutive days. Thus, habituation to rotarod test was parallel with habituation to oral gavage (Figure 1). Each day of training consisted of 3 sessions, with a 120 s rest period between trials. Animals were placed on the rotarod and timed until they fell from the rotating rod. Following last trial, the blood measurements were collected within 10 min.

### 2.6. Blood Glucose and R-βHB

Whole blood (~10 μL) was acquired from saphenous vein (rats) and tail vein (mice) for analysis of blood glucose (mg/dL) and R-βHB (mmol/L) with Precision Xtra^TM^ (Abbott Laboratories, Abbott Park, IL, USA). Note that the Precision Xtra^TM^ measures R-βHB exclusively—not S-βHB, AcAc, or Acetone—therefore, total ketone level may be somewhat underrepresented. R/D and S/L are two stereoisomers, called enantiomers, that are mirror images of each other. While *R*-βHB is the normal product of human and mouse metabolism, it is metabolized much faster than S-βHB is. *S*-βHB is not a normal product of human metabolism, however, it is a transient intermediate of β-oxidation of fatty acids, therefore, administration of the same amount of S-βHB may lead to higher level and more sustained blood levels of S-βHB, compared to similar administration of R-βHB [42]. Blood was drawn prior to the beginning of the treatments (5th day of habituation), and this value was used as the established baseline. Treatment-generated changes in glucose and R-βHB were measured (60 min) after the beginning of treatment (acute treatments, SPD rats). Blood was drawn after treatment started, at either 1 h, 24 h, or after 7 daily treatments (subchronic treatments, SPD, and/or WR rats). During chronic treatments (G1D mice), blood was drawn before treatment started (baseline) and at week 3, 6, and 10 after beginning treatment.

### 2.7. Statistics

Data are presented as the mean ± standard error of the mean (SEM). The effects of ketogenic agents on both R-βHB and glucose, as well as motor performance (latency to fall: rotarod) were compared to experimental controls and respective baseline levels. GraphPad Prism (6.0a) was used for data analysis. Comparisons of data were made using a one or two-way ANOVA with Tukey’s multiple comparisons. Results were considered significant when *p* values were less than 0.05. Results are indicated on figures using the following notations *: *p* < 0.05, **: *p* < 0.01, ***: *p* < 0.001, and ****: *p* < 0.0001 level of significance.

## 3. Results

### 3.1. Changes in Motor Performance, Blood Glucose, and R-βHB Levels in SPD Rats with Acute Exposure

**Figure 2:**
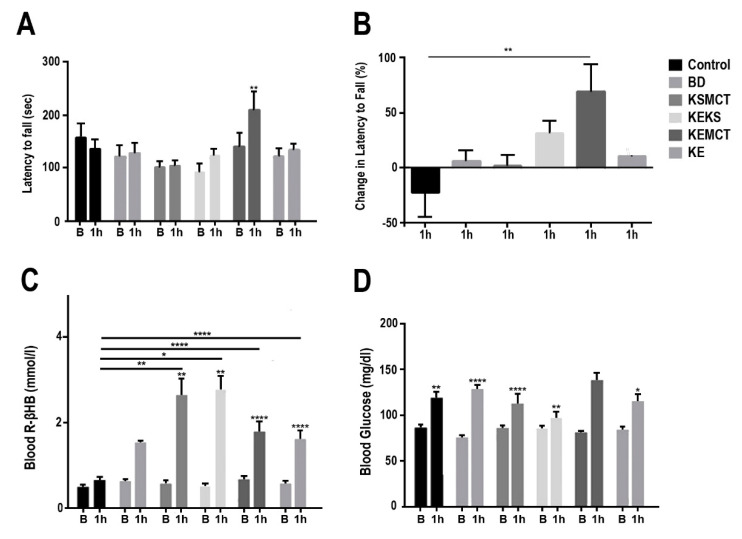
Changes in motor performance and blood parameters from postexercise state for one-year old Sprague-Dawley rats, 1 h following treatment. (**A**) Latency to fall (sec) on accelerating rotarod at each timepoint. (**B**) Percent change in latency to fall on accelerating rotarod at 1 h, compared to baseline. (**C**) Blood R-βHB levels in postexercise state at each timepoint. (**D**) Blood glucose levels in postexercise state at each timepoint. Abbreviations: B: Baseline; 1 h: 1 h timepoint; R-βHB: R-beta-hydroxybutyrate; BD: butanediol; KE: ketone ester; KEKS: combination of ketone ester and ketone salt (KS); KEMCT: ketone ester in combination with medium chain triglyceride (MCT); KSMCT: ketone-salt in combination with medium chain triglyceride (MCT); SPD: Sprague-Dawley rats. *: *p* < 0.05, **: *p* < 0.01, and ****: *p* < 0.0001 level of significance.

In acutely exposed one-year-old SPD rats, the latency to fall from an accelerating rotarod was significantly elevated in KEMCT group, compared to baseline (*p* = 0.0011, Figure 2A). The KEKS group showed a trend of increase, however, the results were nonsignificant, while the BD, KSMCT, and KE groups also showed no significant change in motor performance. The percent change in latency to fall was significantly higher in KEMCT group, compared to control (*p* = 0.003, Figure 2B). Blood R-βHB levels in postexercise state were elevated significantly in KSMCT group, compared to baseline (*p* = 0.0099) and control (*p* = 0.0040), in KEKS group, compared to baseline (*p* = 0.0021) and control (*p* = 0.0119), in KEMCT group, compared to baseline (*p* < 0.0001) and control (*p* < 0.0001), and in KE group, compared to baseline (*p* < 0.0001) and control (*p* < 0.0001, Figure 2C). The BD group displayed a nonsignificant increase in blood R-βHB levels. The blood glucose levels in postexercise state were elevated significantly after 1 h in the control group (*p* = 0.0064), in BD group (*p* < 0.0001), in KSMCT group (*p* < 0.0001), in KEKS group (*p* = 0.0048), and KE group (*p* = 0.0255), compared to their respective baselines (Figure 2D).

### 3.2. Changes in Motor Performance, Blood Glucose, and R-βHB Levels in SPD Rats with Subchronic Exposure

**Figure 3:**
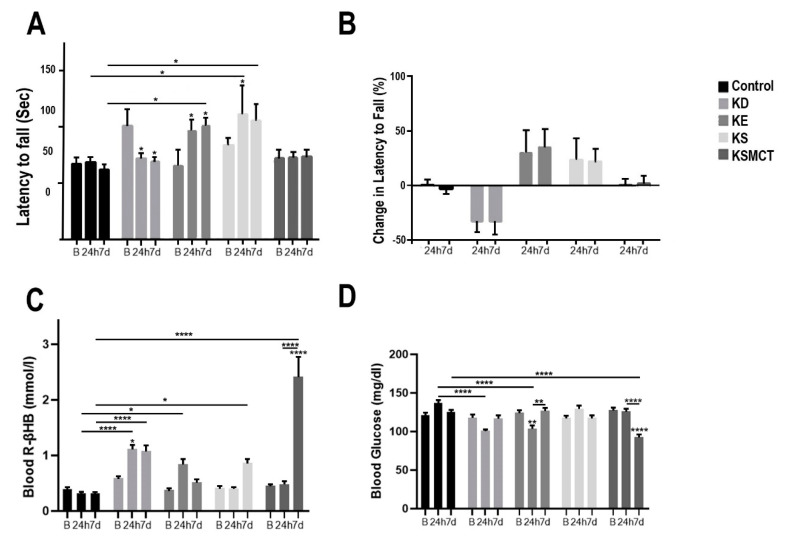
Motor performance and blood parameters in postexercise state for 4-month-old Sprague-Dawley rats after subchronic treatment, measured at baseline, 24 h and at 7 days following treatment. (**A**) The latency to fall (sec) on the accelerating rotarod was presented at each timepoint. (**B**) The percent change in latency to fall on the accelerating rotarod presented at 24 h and at 7 days, compared to baseline. (**C**) Blood R-βHB levels are presented in postexercise state at each timepoint. (**D**) Blood glucose levels are presented in postexercise state at each timepoint. Abbreviations: B: Baseline; 24 h: 24 h timepoint; 7d: 7 days timepoint; R-βHB: R-beta-hydroxybutyrate; KD: ketogenic diet; KE: ketone ester; KS: ketone salt; KSMCT: ketone salt in combination with medium chain triglyceride (MCT); SPD: Sprague-Dawley rats. *: *p* < 0.05, **: *p* < 0.01, and ****: *p* < 0.0001 level of significance.

In the 4-month-old SPD rats after subchronic treatment the latency to fall from the accelerating rotarod was significantly decreased for the KD group at 24 h (*p* = 0.0296) and at 7 days (*p* = 0.0133), compared to baseline (Figure 3A). The latency to fall increased in KE group at 24 h, compared to baseline (*p* = 0.0313), and at 7 days, compared to the control (*p* = 0.0413) and baseline (*p* = 0.0106). The latency to fall increased in KS group at 24 h, compared to the control (*p* = 0.0138) and baseline (*p* = 0.039), and at 7 days, compared to the control (*p* = 0.011). The KSMCT group displayed no significant change in latency to fall. No groups had significantly different percent change in latency to fall, compared to baseline or control (Figure 3B). Blood R-βHB levels increased significantly in the KD group at 24 h, compared to baseline (*p* = 0.038) and control (*p* < 0.0001) and in the KE group at 24 h, compared to control (*p* = 0.0325, Figure 3C). After 7 days the blood R-βHB levels increased significantly in KD and KS groups, compared to the control (*p* < 0.0001 and *p* = 0.0194 respectively), and in KSMCT, compared to baseline (*p* < 0.0001), 24 h (*p* < 0.0001), and control (*p* < 0.0001). Blood glucose level at 24 h was significantly decreased in KD group, compared to control (*p* < 0.0001), and in the KE group, compared to control (*p* < 0.0001) and baseline (*p* = 0.0047, Figure 3D). After 7 days of treatment, the postexercise blood glucose level was decreased in KSMCT group, compared to baseline (*p* < 0.0001), 24 h (*p* < 0.0001), and control (*p* < 0.0001). An increase in blood glucose level was observed in the KE group relative to its 24-h reading (*p* = 0.0049).

### 3.3. Changes in Motor Performance, Blood Glucose, and R-βHB Levels in WR Rats with Acute and Subchronic Exposure

**Figure 4:**
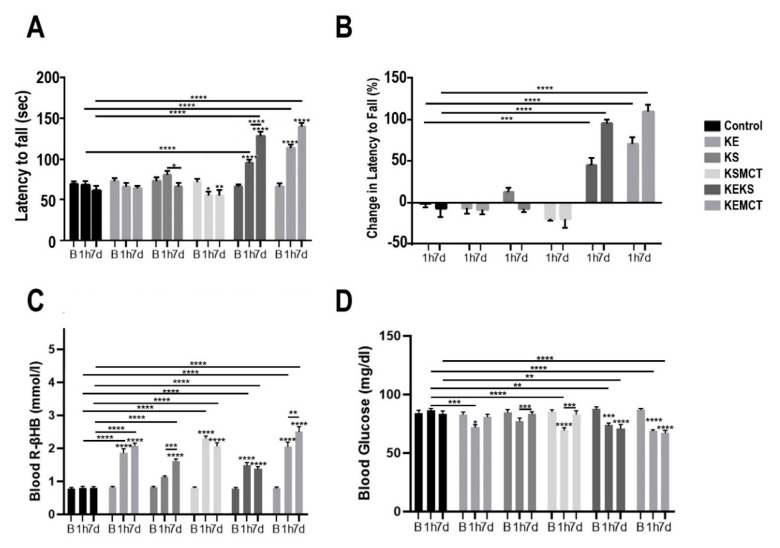
Changes in motor performance and blood parameters in postexercise state for WR rats with acute and subchronic exposure, at baseline, at 1 h and 7 days following treatment. (**A**) The latency to fall (sec) on accelerating rotarod at each timepoint. (**B**) The percent change in latency to fall on accelerated rotarod, compared to baseline at 1 h and 7 days post treatment. (**C**) Blood R-βHB levels are presented in postexercise state at each timepoint. (**D**) Blood glucose levels are presented in postexercise state at each timepoint. Abbreviations: B: Baseline; 1 h: 1 h timepoint; 7d: 7 days timepoint; R-βHB, R-beta-hydroxybutyrate; KE: ketone ester; KEKS: combination of ketone ester and ketone salt (KS); KEMCT: ketone ester in combination with medium chain triglyceride (MCT); KS: ketone salt; KSMCT: ketone-salt in combination with medium chain triglyceride (MCT); WR: WAG/Rij rats. *: *p* < 0.05, **: *p* < 0.01, ***: *p* < 0.001 and ****: *p* < 0.0001 level of significance.

In WR rats with acute and subchronic exposure, the latency to fall on accelerating rotarod was significantly decreased in KSMCT group at 1 h and at 7 days, compared to the baseline (*p* < 0.0129; *p* = 0.0078; respectively, Figure 4A). In KEKS group the latency to fall increased at 1 h, compared to baseline (*p* < 0.0001) and control (*p* < 0.0001), and at 7 days, compared to baseline (*p* < 0.0001), 1 h (*p* < 0.0001), and control (*p* < 0.0001). In the KS group the latency to fall significantly decreased between 1 h and 7 days (*p* = 0.0138). The latency to fall increased in KEMCT group at 1 h, compared to baseline (*p* < 0.0001) and control (*p* < 0.0001), and at 7 days, compared to baseline (*p* < 0.0001) and control (*p* < 0.0001). The percent change in latency to fall on the accelerated rotarod was significantly increased in the KEKS group at 1 h, compared to control (*p* = 0.0001), and at 7 days, compared to control (*p* < 0.0001, Figure 4B). The KEMCT group at 1 h had a significantly increased percent change in latency to fall, compared to control (*p* < 0.0001) and similarly at 7 days, compared to control (*p* < 0.0001).

Blood R-βHB levels were increased significantly after 1 h in the KE, KSMCT, KEKS, and KEMCT groups, compared to their respective baselines (*p* < 0.0001) and control (*p* < 0.0001, Figure 4C). All treatment groups at 7 days had elevated blood ketone levels, compared to their baselines (*p* < 0.0001) and the control (*p* < 0.0001). At 7 days, the blood ketone levels in KEMCT and KS groups were significantly increased from their 1-h level (*p* = 0.0025; *p* = 0.0007; respectively). Blood glucose levels decreased significantly at 1 h in the KE group, compared to baseline (*p* = 0.0307) and control (*p* = 0.0002), in the KSMCT group, compared to baseline (*p* < 0.0001) and control (*p* < 0.0001), in the KEKS group, compared to baseline (*p* = 0.0003) and control (*p* = 0.0022), and in KEMCT group, compared to baseline (*p* < 0.0001) and control (*p* < 0.0001, Figure 4D). After 7 days, blood glucose levels decreased in KEKS group, compared to baseline (*p* < 0.0001) and control (*p* = 0.0034), increased in KS and KSMCT groups, compared to 1 h (*p* = 0.0006; *p* = 0.0006), and decreased in KEMCT group, compared to baseline (*p* < 0.0001), and control (*p* < 0.0001).

### 3.4. Changes in Motor Performance, Blood Glucose, and R-βHB Levels in G1D Mice with Chronic Exposure

**Figure 5:**
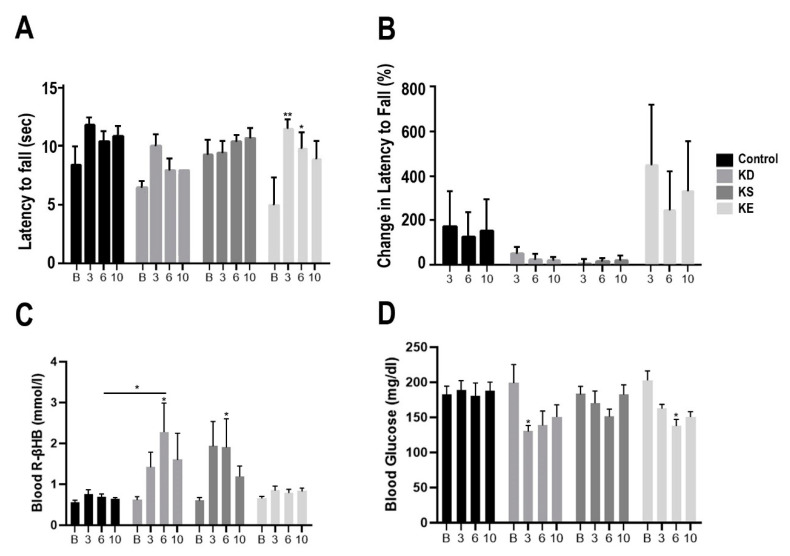
Changes in motor performance and blood parameters in postexercise state for G1D mice with chronic treatment, at baseline, at week 3, at weeks 6, and at week 10. (**A**) The latency to fall (sec) on accelerating rotarod presented, compared to baseline. (**B**) The percent change in latency to fall at each timepoint, compared to baseline. (**C**) Blood R-βHB levels are presented in postexercise state at each timepoint. (**D**) Blood glucose levels are presented in postexercise state at each timepoint. Abbreviations: B: Baseline; 3: Week 3; 6: Week 6; 10: Week 10; R-βHB, R-beta-hydroxybutyrate; G1D, Glucose Transporter Type-1 Deficiency Syndrome mice; KD, ketogenic diet; KE, ketone ester; KS, ketone-salt. *: *p* < 0.05, **: *p* < 0.01 level of significance.

In G1D mice after chronic treatment the latency to fall on the accelerating rotarod was significantly increased in the KE group at week 3 (*p* < 0.01), and at week 6 (*p* < 0.05), compared to baseline (Figure 5A). The KD and KS groups were observed to have a trend of nonsignificant increase in latency to fall. The percent change in latency to fall was not significantly elevated in any groups, compared to baseline or control (Figure 5B). KD and KS were observed to have a marginal and nonsignificant increase percent change in latency to fall, while the KE had a larger, but still nonsignificant increase in percent change in latency to fall. Blood R-βHB levels increased significantly in the KD group at week 6, compared to baseline (*p* = 0.0157) and control (*p* = 0.0211, Figure 5C). The KS group had a significant increase in blood R-βHB levels at week 6, compared to baseline (*p* = 0.045). Blood glucose level decreased significantly in the KE group at week 6, compared to baseline (*p* = 0.0113), and in the KD group at week 3, compared to baseline (*p* = 0.0393, Figure 5D).

## 4. Discussion

In this study, we demonstrated the effect of KD and various exogenous ketone formulations on motor performance in different rodent models with (WR rats and G1D mice) and without (SPD rats) pathology after acute, subchronic, or chronic exposures. We reported improvement of motor performance by ketone supplementation, which may depend on the strain of rodents, specific ketone formulation, age, species, and exposure frequency. Moreover, we reported significant changes in blood *R*-βHB and glucose levels after exercise. Previous experiments have demonstrated that ketogenic diets significantly improved motor function in studies on AD, ALS, HD, PD, stroke, and MS disease models [1,2,3,4,5,6,7,9,33]. Our experiments expand upon these previous studies by reporting about the positive effect of exogenous ketone supplementation on motor performance in nonpathological and additional pathological rodent models.

Ketone-based therapies were found to be effective in the treatment of a variety of diseases, including neurological pathologies with motor dysfunction [2,8,25,43,44,45,46,47,48,49,50,51,52,53,54]. For example, in patients with PD nutritional ketosis decreased neuron degeneration, reduced mitochondrial damage, improved motor outcomes, and reduced proinflammatory cytokine levels [1,4,43,44,45,46]. Application of nutritional ketosis attenuated motor dysfunction in mouse models of HD [47]. Ketone-based therapies were reported effective in improving motor function in a model animal of ALS (SOD1-G93A transgenic mice) through neuroprotective outcomes [2,35]. Moreover, in a study on G1D mice (characterized by impaired glucose transportation and motor dysfunction), adherence of a KD improved motor symptoms [41]. Our results demonstrated that exogenous ketone supplement can improve motor dysfunction in G1D mice. While several therapeutic mechanisms for the KD have been explored, its role in disease pathologies is relatively unknown and further research into the physiological and mechanistic effect of ketone-based therapeutics is warranted [1]. It has been demonstrated that exogenous ketone supplementation is a viable and safe method in various animal models of, for example, central nervous system (CNS) diseases and in human CNS disorders when alternative ketone-based therapies are needed that address the limitations with KD [1,14,17,19,31].

We report that the motor performance may improve with KD in disease context, but also with exogenous ketone supplementation even in rodents eating a standard, carbohydrate-rich diet, suggesting that ketone supplementation may be a viable alternative for those unwilling or unable to adhere to a KD. Our data supports that exogenous ketone supplementation was more efficient in improving motor performance than KD, as KD reduced motor performance in SPD rats after 24 h and 7 days (Figure 3A), although the effect was not significant when assessing % change (Figure 3B). On the other hand, exogenous ketones showed more consistent improvement throughout different model systems, illustrating a potentially more translatable therapeutic tool across healthy and disease context.

While our previous study showed that ketone supplementation lowered blood glucose levels [27] in the nonexercised state, we found that in the postexercise state it was elevated at 1 h (Figure 2D), possibly due to exercise augmenting glycogenolysis induced glucose elevation which may offset the blood glucose lowering effect. However, in some cases in SPD and WR rats after 1 h and 7 days the blood glucose levels were lower than control and baseline (Figure 2D, Figure 3D and Figure 4D) and in G1D mice after 6 weeks it was lower than baseline (Figure 5D), suggesting that exogenous ketones may be able to offset the glycogenolysis induced glucose elevation in postexercised state. It has been suggested that alleviating effects of ketone-based therapies, such as KD on motor performance, likely results from carbohydrate restriction (and low glucose levels), but not from increased blood βHB levels [48,49]. However, based on our results it is unclear whether KD- and ketone supplements- evoked alterations in blood glucose level had a major influencing factor on motor performance. For example, both KD and KE decreased blood glucose level at 24 h (Figure 3D), but only KE improved motor performance of SPD rats.

Many different beneficial effects on diseases have been linked to ketone therapies and this is likely due to the resulting decreased production of reactive oxygen species (ROS), improved mitochondrial function, reduction in inflammation, neuroprotective effects, and increased expression of brain-derived neurotrophic factor (BDNF) [1,50,51,52]. Additionally, ketones have been found to produce changes in post-translational modification of proteins and histone acetylation [53,54,55]. It has also been reported that ketones may enhance learning in multiple rodent models and may explain the motor performance increase observed in the nonpathological mouse models [56]. Furthermore, ketones may enhance muscle performance or CNS control of muscle contractions/coordination, which could be a contributing factor to the rodent’s ability to remain on the rotarod longer, not only in rodents with motor dysfunction, but also rodents without motor impairment, such as SPD rats and WR rats.

Ketogenic diet and exogenous ketone supplementation may also act on cellular homeostasis through various signaling pathways, such as mTOR, AMPK, and neurotransmitter systems. Ketosis has been shown to affect cell proliferation, energetic metabolism, protein biosynthesis, and attenuate muscle wasting [57,58,59,60,61,62]. KD acts upon specific tyrosine kinase receptors for insulin and insulin growth factor (IGF) and upregulates the phosphatidylinositol-3 kinase (PI3K)-Akt-mammalian target of rapamycin complex 1 (mTORC1). This is counteracted by a lower intracellular ATP/AMP ratio and the subsequent upregulation of liver kinase B1 (LKB1)-AMP-activated protein kinase (AMPK) signaling, which then inhibits the mTORC1 pathway [48,53]. Research also suggests that ketone-based therapies likely have additional therapeutic applications by reduced levels of proinflammatory cytokines, ROS, attenuation of skeletal muscle catabolism, and various neuroprotective effects [15,50,51,62,63]. Moreover, it was also suggested that KD- and exogenous ketone supplements-evoked alleviating effects on different CNS diseases may be modulated by different neurotransmitter/neuromodulator systems, such as GABAergic (e.g., by increased GABA level) and purinergic/adenosinergic (e.g., via increased adenosine concentration) systems [19,31,64,65,66,67]. Therefore, studies about the application of ketone metabolic therapies for various neurological conditions suggest that multifactorial mechanisms play a role in improving motor function (e.g., mitochondrial function, neurotransmitter systems, anti-inflammatory processes, and skeletal muscle physiology), but further research is required to fully elucidate pathways and potential therapeutic applications.

While no motor function impairments (motor dysfunction) have been demonstrated in SPD rats and WR rats [39,40], the subchronically administered ketone supplement KE, KS (in SPD rats), KEKS and KEMCT (in WR rats) improved motor performance. These results suggest that exogenous ketone supplements may improve motor performance not only in animal models with neurometabolic pathology, such as motor dysfunction (e.g., G1D mice), but also in animals without pathology (e.g., SPD rats) or with pathology, but without motor dysfunction (e.g., WR rats), likely by enhanced learning and muscle performance [5,44,56,68]. Moreover, exogenous ketone supplements-evoked effects on motor performance may be rat strain dependent. Indeed, it was demonstrated that influences of KD and ketone supplements may be modulated by strain-, age-, and species-dependent changes. For example, it has been demonstrated that expression of monocarboxylate class type 1 transporter on the BBB may be not only age-, but also species-dependent [69,70,71]. In addition, age- and species-dependent changes in activity of βHB metabolizing enzymes were also demonstrated [39,40,72,73,74,75]. It has been revealed that level, uptake, metabolism, as well as utilization, and consequently, effects of ketone bodies may be regionally different in the brain areas implicated not only in physiological, but also in pathophysiological processes in different strains [72,76,77,78]. All of these factors may result in brain area-, age-, strain-, and species-dependent influences/effectivity of KD and ketone supplements on motor performance in different animal models. Consequently, efficacy of treatments may also be dependent on the various type of ketone supplements (e.g., KSMCT decreased whereas KEKS and KEMCT increased the motor performance in WR rats), on the exposure frequency/time after administration (e.g., acute treatment by KE was not effective on motor performance, but subchronic administration of KE improved the motor performance in SPD rats) and on the interference of different factors.

### Limitations and Inconsistencies

One limitation of these results is that we used racemic (R/S) βHB exclusively, because this was the most economical and commercially available product on the market for both experimental purposes and for consumer consumption at the time of the study. Racemic βHB has been used clinically in patients and consumers for years [79], and no serious adverse events from βHB supplement ingestion have been reported (CAERS open-FDA database), even with >1 million estimated servings consumed annually. Thus, our approach was to evaluate molecules, which may be economically feasible to translate to humans and would be accessible to end-users in the near future. However, one consideration with racemic βHB supplements is the potential differences in metabolism of S- βHB and R- βHB, although existing data on this is still limited. Based on previous studies we know that S- βHB and R- βHB from racemic mixtures are naturally metabolized in rats and humans [80], but S-βHB remains elevated in the blood longer after consumption of racemic mixtures. It appears that ketolytic metabolism favors a more rapid catabolism of R- βHB, the predominant form of βHB that is produced endogenously. It is possible that the rate limiting enzymes for S-βHB conversion to acetyl CoA (e.g., S-3-hydroxybutyryl-CoA dehydrogenase) may be in lower quantities since endogenous levels are much lower. Although previous studies suggest that the enzymatic kinetics of S- βHB metabolism is slower, this does not appear to be the case in the rat liver, at least with S-1,3-butanediol. Of potential relevance to our results is the observation that S-1,3-butanediol, a specific S- βHB precursor, has a greater blood glucose lowering effect [81], which could conceivably influence motor function performance. These previous metabolic observations need to be considered when interpreting our data and designing future experiments that test enantiomerically pure forms of both βHB isomers in physiological testing.

Considering these limitations, we can speculate that R-βHB levels may be an important factor in KD/exogenous ketone supplement-evoked positive effects on motor performance, but other factors likely have a modulatory role. Since we could not unambiguously correlate elevated R-βHB levels with augmented motor performance, future dose-response studies can help to identify the optimal range for this specific application. It is also possible that other forms of ketone molecules contributed to the performance enhancing effect that were not directly measured in the present study, but previous studies show their elevation (e.g., KE will elevate acetoacetate levels as well, which was not directly measured here, but may have contributed to the performance enhancing effect, see Figure 5B,C). For example, acute KSMCT and KEKS treatment significantly increased the blood R-βHB level at 1 h, but did not augment motor performance (latency to fall), whereas KEMCT treatment enhanced R-βHB concentration to a lesser degree (compared to KSMCT and KEKS), but significantly increased latency to fall, compared to baseline in one year old SPD rats (Figure 2). Acetoacetate, from KE, may have contributed to the performance enhancing effect here as well, which may have increased efficacy for this application when working synergistically with MCT rather than with KS. In regards to KD treatment, we observed increased blood R-βHB at 24 h and 7 days, but this treatment significantly decreased the latency to fall from rotarod, compared to baseline in 4-months-old SPD rats (Figure 3). It is likely that adaptation (which takes weeks to months) to the KD confers additional beneficial effects, so this outcome was not unexpected after only 24 h and even 7 days, as we would expect to see ketoadaptation and its beneficial effects over longer timeframe. KE treatment significantly increased blood R-βHB level only at 24 h, but improved motor function at both 24 h and 7 days, compared to baseline (24 h and 7 days), and control (at 7 days). KS treatment did not increase blood R-βHB levels at 24 h, however this treatment significantly improved motor function, compared to both control and baseline (Figure 3). It is conceivable that low R-βHB at 24 h is indicative of tissue utilization, leading augmentation of muscle tissue performance. Moreover, KSMCT treatment increased blood R-βHB concentration to high levels at 7 days more than any other treatment, but it did not show a motor performance improving effect (Figure 3), suggesting that higher R-βHB levels may not be as beneficial, or that MCT may be negating the performance benefits (perhaps by reducing R-βHB metabolism). Subchronic treatment of WR rats by KE and KSMCT increased blood R-βHB levels at 1 h and at 7 days, but did not improve motor performance (Figure 4), again suggesting that higher R-βHB levels might not be optimal in this context. Chronic KE treatment improved motor performance at week 3 and week 6 in G1D mice, but blood R-βHB levels were not changed by KE treatment at these time points (Figure 5). Therefore, it is possible that other ketone bodies, such as acetoacetate may contribute to the performance enhancing effect that was associated with chronic KE treatment. Based on these results, we can speculate that a particular range of R-βHB level may be needed to improve motor function, and this may be age-, strain-, and species dependent. It is possible that higher levels of acute (or subchronic) ketosis may induce a mild metabolic acidosis (lower pH), and this could conceivably blunt performance effects. Moreover, other contributing factors should also be considered, e.g., different ketone-based therapies evoke direct and indirect influences on neurotransmission, cell energetics, muscle and nerve regeneration, as well as gastric hormone levels (e.g., ghrelin, which is in connection with muscular trophism) [48,82,83], other modulatory effects, such as mobilization of polyunsaturated fatty acids (PUFAs),which can directly modulate ion channel functioning [84,85], and elevated neurosteroid synthesis resulting in modulation of GABAA receptors [86,87]. These and other, yet unidentified factors may influence the modulatory role of KD/ketone supplements on physiological and pathophysiological processes, as well as CNS diseases. Thus, our results suggest that KD/ketone supplements may be effective to improve motor function in different physiological and pathophysiological conditions by affecting multiple organs.

Given these inconsistencies we need to point out the following: (i) we could not make definitive conclusion about direct correlation between motor performance and R-βHB levels; (ii) our experimental design did not mechanistically elucidate the contributing role of acetoacetate (and perhaps acetone) in augmenting motor performance; (iii) these data do not clarify if ketone supplementation augments performance via enhancing muscular cellular energetics or through some yet to be identified ketone-induced signaling/neuropharmacological change, potentially influencing CNS-control of motor performance. However, we can conclude that (i) positive effects of exogenous ketone supplementation were identified on motor performance in nonpathological and pathological states; (ii) exogenous ketones may be able to offset the glycogenolysis induced glucose elevation in postexercised state; (iii) exogenous ketones may have a specific therapeutic range for various applications; (iv) different forms and formulations can have different effects independently from the level of blood βHB elevation.

In order to address these remaining questions, future experiments will need to focus on: (1) More direct comparisons of the physiological effects between racemic and enantiomerically pure βHB substances; (2) Comprehensively evaluate the dose-dependent effects of both racemic and enantiomerically purified forms of βHB; (3) Investigate acetoacetate-specific effects; (4) Monitoring correlations between blood glucose lowering effect and motor performance; (5) Conduct additional tests focusing on potential differences in efficacy between various motor functions, e.g., endurance, fine motor control, strength, balance; (6) Mechanistically isolate R-βHB signaling effects from ketone-induced changes in cellular energetics and metabolic control, especially focusing on signaling effects that can influence motor performance; (7) Detailed investigation of agent-specific mechanism on not only the brain, but also on other organs (e.g., heart, skeletal muscle, lungs, and liver); (8) Develop and identify optimal ketone formulations (e.g., types, doses, exposure frequency vs. age) to alleviate motor dysfunction in patients with different physiological and pathological conditions.

In light of these inconsistencies in the data we would like to caution end-users to avoid making generalizations about the use of ketone supplements in different contexts and we call to direct more attention to the differences between various forms (racemic and enantiomerically pure) and formulations (combined agents) and their potential positive, negative, or synergistic effect.

## 5. Conclusions

Our study demonstrates the effects of the KD and ketone supplementation on motor performance in SPD, and WR rats, as well as in G1D mice after acute, subchronic, or chronic administration. We observed that motor performance improved significantly in various pathological and nonpathological rodent models for different exposure schedules and that the effectiveness of the supplements differed according to rodent strain, the schedule of treatment, the specific supplement they were exposed to, and age of the rodents. We found that exogenous ketone supplementation was more efficient in improving motor performance than KD. While KD treatment led to mixed results—demonstrating more efficacy in disease context (G1D)—exogenous ketones showed consistent effect across disease and nondisease states. Furthermore, in certain scenarios, exogenous ketone supplements offset the glycogenolysis-induced glucose elevation in the postexercise state, which might be beneficial when there is a need to keep blood glucose levels low even after exercising. These results also strengthen previous research on the potential of ketone therapies and their ability to improve motor performance in a variety of pathological and nonpathological rodent models. The differences in the efficacy of a given formulation are potentially due to differences in neuronal ketone metabolism between the different rodent strains and species, hepatic metabolism (ketogenesis), or variabilities in skeletal muscle metabolism. However, further studies are needed to explore the safe and effective therapeutic applications of ketone-based therapies and elucidate their exact mechanisms of action on motor performance.

## Figures and Tables

**Table 1:** Macronutrient information of each diet. (More details about the ingredients of each diet can be found in Supplementary Tables S1 and S2).

	Standard Diet (SD)	Ketogenic Diet (KD)
Protein, % of kcal	24	22.4
Carbohydrate, 5 of kcal	62	0.5
Fat, % by kcal	14	77.1
kcal/g	3.0	4.7

## Supplementary Materials

The following are available online at https://www.mdpi.com/2072-6643/12/8/2459/s1, Table S1: NIH-31 Open Formula Mouse/Rat Sterilizable Diet, Table S2: Teklad Custom Research Diet Data Sheet.
