# Supplementary material for: Exogenous Ketone Supplements Improved Motor Performance in Preclinical Rodent Models
Source: Nutrients. 2020 Aug 15;12(8):2459. doi: 10.3390/nu12082459 (PMC7468837; doi:10.3390/nu12082459)
Supplement: Supplementary file 1 [file nutrients-12-02459-s001.zip › Supplementary material1.pdf]

7017

NIH-31 Open Formula Mouse/Rat Sterilizable Diet

Product Description- 7017 is a fixed and open formula, autoclavable diet manufactured with high quality ingredients and designed to support maintenance, growth, and reproduction of rodents. 7017 is supplemented with additional vitamins to ensure nutritional adequacy after autoclaving. **Also available certified (7017C) and irradiated (7917).**

Ingredients (in descending order of inclusion)- Ground wheat, ground corn, ground oats, wheat middlings, fish meal, dehulled soybean meal, dehydrated alfalfa meal, corn gluten meal, soybean oil, dicalcium phosphate, brewers dried yeast, calcium carbonate, iodized salt, choline chloride, magnesium oxide, kaolin, ferrous sulfate, menadione sodium bisulfite complex (source of vitamin K activity), manganous oxide, thiamin mononitrate, niacin, calcium pantothenate, vitamin E acetate, vitamin A acetate, copper sulfate, zinc oxide, riboflavin, pyridoxine hydrochloride, vitamin B_{12} supplement, vitamin D_3 supplement, calcium iodate, biotin, folic acid, cobalt carbonate.

Standard Product Form: ***Pellet***

Macronutrients

Crude Protein	%	18.0
Fat (ether extract) [a]	%	4.7
Carbohydrate (available) [b]	%	46.5
Crude Fiber	%	4.0
Neutral Detergent Fiber [c]	%	13.6
Ash	%	6.2
Energy Density [d]	kcal/g (kJ/g)	3.0 (12.6)
Calories from Protein	%	24
Calories from Fat	%	14
Calories from Carbohydrate	%	62

Minerals

Calcium	%	1.1
Phosphorus	%	1.0
Non-Phytate Phosphorus	%	0.7
Sodium	%	0.3
Potassium	%	0.6
Chloride	%	0.5
Magnesium	%	0.2
Zinc	mg/kg	47
Manganese	mg/kg	155
Copper	mg/kg	13
Iodine	mg/kg	2
Iron	mg/kg	270
Selenium	mg/kg	0.30

Amino Acids

Aspartic Acid	%	1.5
Glutamic Acid	%	3.2
Alanine	%	1.1
Glycine	%	1.0
Threonine	%	0.7
Proline	%	1.5
Serine	%	0.9
Leucine	%	1.4
Isoleucine	%	0.8
Valine	%	0.8
Phenylalanine	%	0.8
Tyrosine	%	0.7
Methionine	%	0.4
Cystine	%	0.3
Lysine	%	0.8
Histidine	%	0.4
Arginine	%	1.0
Tryptophan	%	0.2

Vitamins

Vitamin A [e, f]	IU/g	24.2
Vitamin D_3 [e, g]	IU/g	4.2
Vitamin E	IU/kg	41
Vitamin K_3 (menadione)	mg/kg	22
Vitamin B_1 (thiamin)	mg/kg	76
Vitamin B_2 (riboflavin)	mg/kg	7
Niacin (nicotinic acid)	mg/kg	87
Vitamin B_6 (pyridoxine)	mg/kg	9
Pantothenic Acid	mg/kg	39
Vitamin B_{12} (cyanocobalamin)	mg/kg	0.06
Biotin	mg/kg	0.30
Folate	mg/kg	2
Choline	mg/kg	1890

Fatty Acids

C16:0 Palmitic	%	0.7
C18:0 Stearic	%	0.1
C18:1ω9 Oleic	%	1.0
C18:2ω6 Linoleic	%	1.9
C18:3ω3 Linolenic	%	0.2
Total Saturated	%	0.9
Total Monounsaturated	%	1.2
Total Polyunsaturated	%	2.1

Other

Cholesterol	mg/kg	50

[a] Ether extract is used to measure fat in pelleted diets, while an acid hydrolysis method is required to recover fat in extruded diets. Compared to ether extract, the fat value for acid hydrolysis will be approximately 1% point higher.

[b] Carbohydrate (available) is calculated by subtracting neutral detergent fiber from total carbohydrates.

[c] Neutral detergent fiber is an estimate of insoluble fiber, including cellulose, hemicellulose, and lignin. Crude fiber methodology underestimates total fiber.

[d] Energy density is a calculated estimate of *metabolizable energy* based on the Atwater factors assigning 4 kcal/g to protein, 9 kcal/g to fat, and 4 kcal/g to available carbohydrate.

[e] Indicates added amount but does not account for contribution from other ingredients.

[f] 1 IU vitamin A = 0.3 μg retinol

[g] 1 IU vitamin D = 25 ng cholecalciferol

For nutrients not listed, insufficient data is available to quantify.

Nutrient data represent the best information available, calculated from published values and direct analytical testing of raw materials and finished product. Nutrient values may vary due to the natural variations in the ingredients, analysis, and effects of processing.

Teklad Diets are designed and manufactured for research purposes only.

© 2015 Envigo

Teklad Diets + Madison WI + envigo.com + tekladinfo@envigo.com + (800) 483-5523
