# Supplementary material for: Exogenous Ketone Supplements Improved Motor Performance in Preclinical Rodent Models
Source: Nutrients. 2020 Aug 15;12(8):2459. doi: 10.3390/nu12082459 (PMC7468837; doi:10.3390/nu12082459)
Supplement: Supplementary file 1 [file nutrients-12-02459-s001.zip › Supplementary material2.pdf]

TD.10911 Ketogenic Diet 2 (MCT, Flax, Canola)

Formula	g/Kg
Casein	300.0
L-Cystine	2.86
Cellulose	245.31
Medium Chain Triglycerides (MCT) Oil	270.0
Flaxseed Oil	70.0
Canola Oil	60.0
Mineral Mix, Ca-P Deficient (79055)	18.5
Calcium Phosphate, dibasic	8.5
Calcium Carbonate	10.75
Vitamin Mix, Teklad (40060)	14.0
Ethoxyquin, antioxidant	0.08

Footnote

Modified from TD.10787 to replace maltodextrin with cellulose to make diet essentially free of carbohydate. Diet has a 2:1 ratio of n-3 to n-6 fatty acids and a 1.5:1 ratio of fat to protein + carbohydrate. Compared to TD.10787, TD.10911 has the same % fat (wt/wt) but has a higher % kcal from fat, because carbohydrate was removed and replaced with cellulose.

Selected Nutrient Information[1]

	% by weight	% kcal from
**Protein**	26.4	22.4
**Carbohydrate**	0.6	0.5
**Fat**	40.3	77.1

Kcal/g 4.7

[1] Values are calculated from ingredient analysis or manufacturer data

Teklad Diets are designed & manufactured for research purposes only.

Speak With A Nutritionist

- **(800) 483·5523**
- **askanutritionist@harlan.com**

Harlan Laboratories · PO Box 44220 · Madison, WI 53744-4220
www.harlan.com

Key Features

- **Purified Diet**
- **Ketogenic Diet (1.5:1 Ratio)**
- **Omega-3 fatty acids & MCT**
- **Very Low Carbohydrate**

Key Planning Information

- *Products are made fresh to order*
- Store product at 4°C or lower
- Use within 6 months (applicable to most diets)
- Box labeled with product name, manufacturing date, and lot number
- Replace diet at minimum once per week

  More frequent replacement may be advised
- Lead time:
  - 2 weeks non-irradiated
  - 4 weeks irradiated

Product Specific Information

- Powder (paste)
- Minimum order 3 Kg
- Irradiation available upon request

Options (Fees Will Apply)

- Rush order (pending availability)
- Irradiation (see Product Specific Information)

International Inquiry

·Outside U.S.A. or Canada·

- **askanutritionist@harlan.com**

Place Your Order (U.S.A. & Canada)

· Place Order · Obtain Pricing ·
· Check Order Status ·

- **(800) 483·5523**
- **(608) 277·2066** *facsimile*
- **tekladinfo@harlan.com**

Helping you do research better

Harlan, Harlan Laboratories, Helping you do research better,
and the Harlan logo are trademarks and trade names of Harlan Laboratories, Inc.
© 2008 Harlan Laboratories, Inc.
